# Lateral meniscal posterior root tears experience acceptable healing status after transtibial repair technique

**DOI:** 10.1186/s40634-021-00433-z

**Published:** 2021-12-09

**Authors:** Cathrine Aga, Ingerid Baksaas Aasen, Carsten Brocker, Nina Jullum Kise, Stig Heir

**Affiliations:** Orthopaedic Department, Martina Hansens Hospital, Baerum, Norway

## Abstract

**Purpose:**

To evaluate patient MRI results, demography and clinical outcome following transtibial repair of lateral and medial meniscal posterior root tears.

**Methods:**

Patients treated with transtibial repairs of posterior meniscal root tears from 2015 through 2018 performed pre- and postoperative MRI scans. Outcome measures were continuity/discontinuity of the meniscal root and change in meniscal extrusion on MRI. Other outcomes were KOOS, Lysholm score, Tegner activity scale and the Global Rate of Change (GRoC) score for function and pain at follow-up.

**Study design:**

Retrospective case-series.

**Results:**

Of 41 patients, 36 attended follow-up at mean 26 (12–38) months postoperatively. At follow-up, 11 out of 18 lateral meniscus posterior root tear (LMPRT) versus 5 out of 18 medial meniscus posterior root tear (MMPRT) repairs were classified as healed. Meniscal extrusion decreased in LMPRTs from of 2.3 ± 1.5 mm to 1.4 ± 1.09 mm (*p* = 0.080) and increased in MMPRTs from 3.1 ± 1.6 mm to 4.8 ± 1.9 mm (*p* = 0.005) at FU (between-group difference, *p* < 0.001). LMPRT repairs were associated with ACL injury and additional meniscal injury and were younger and with lower BMI. No between-group differences were found for KOOS, Lysholm or GRoC Function scores. Tegner scale was higher and GRoC Pain score lower in the LMPRT group compared to the MMPRTs.

**Conclusion:**

Following transtibial repair for meniscal posterior root repairs, the LMPRTs had a higher frequency of healing, whereas most MMPRTs continued to extrude, despite surgical intervention. The study confirmed that LMPRTs and MMPRTs differ in demography and associated injuries.

## Background

Posterior root tears of the medial and lateral meniscus are known to have devastating consequences for the knee if left untreated [1, 4, 9, 32]. Currently, the transtibial meniscal root repair is the surgical treatment of choice and with this technique the intraarticular contact pressures and tibiofemoral contact areas can be restored [8, 10, 23, 30]. Although the surgical treatment options are identical for both medial and lateral meniscal posterior root tears, these two conditions arise in different populations and are associated with different underlying factors [13, 20]. Clinical studies have shown acceptable outcomes after repair for both conditions [5, 17, 24].

MRI scans of the affected knees can be used to evaluate the degree of healing of the posterior root repairs [12, 31]. Both continuity of the meniscal root at footprint and the meniscal extrusion out of the tibiofemoral joint are correlated with the prognosis and patient related outcome after repair [8, 9, 27]. Whether medial and lateral posterior meniscal root repair show a difference in their aspects of healing, is of interest when considering treatment options for these kinds of injuries. There are relatively few studies published on the outcome after transtibial root repair of medial and lateral menisci, and no study compare MRI results between the two conditions [20].

The primary purpose of this study was to verify the degree of healing and the degree of meniscal extrusion in the two groups LMPRTs and MMPRTs after transtibial root repair. Secondary purposes were to look at demography and clinical outcomes of the same two groups. Our hypothesis was that there would be a difference in the potential for healing and change in meniscal extrusion after repair of LMPRT compared to repair of MMPRT as evaluated by MRI at a minimum of 1 year follow-up.

### Study design

Retrospective case-series, Level IV.

## Material and methods

The study was performed at a single hospital specialized in orthopedic surgery. The hospital administrative system was used to retrospectively identify patients treated with transtibial repair of medial or lateral meniscal root tears. This treatment was first implemented at the hospital in 2015 and all patients exposed to this procedure during April 2015 through June 2018 were invited to attend to the study. Patients that were not able to understand and speak Norwegian language or not able/willing to meet to the hospital for a clinical examination were excluded from the study. Patients having additional ligament injury of the MCL, PCL, PLC or LCL or simultaneous alignment corrections (osteotomy) procedures, were also not included in the study. Patients with Kellgren-Lawrence (KL) grade three or four at preoperative radiographs or ICRS score three or four at surgery were not considered for root repair.

All study participants received information about the study and signed a consent. Information and details from the surgical procedures including concurrent intraarticular pathology were extracted from the patients’ journals. Follow-up clinical examination were performed by two experienced orthopedic surgeons together with a physiotherapist. All patients were referred to their nearest radiographic institute to perform a new MRI scan at follow-up. Pre- and postoperative MRI scans were examined and evaluated by a radiologist specialized in musculoskeletal radiology. The methodology of evaluating the MRIs were discussed between the authors based upon a literature search [18, 28, 31]. Location, appearance and continuity/discontinuity of the fibers and the degree of extrusion were recorded [25]. To ensure the reliability of the findings, the inter-rater reliability (ICC) between three observers was measured.

### Surgery

Anteromedial and anterolateral portals were established, a standard arthroscopy of the joint was performed and the posterior roots of the menisci were evaluated. If a posterior meniscal root tear was present, the transtibial fixation technique was performed in a standardized approach [3, 7, 22]: Additional portals and cannulas were established if necessary. The footprint location for root attachment was prepared with curette and shaver. An anatomic footprint was aimed for [14]. The root was loosened from adherent scar tissue if necessary, and the meniscofemoral ligament preserved if present. Sutures were thread through the meniscal root with a suture machine and although two sutures were aimed for, different suture techniques and materials were used. An external aiming drill-guide was used to drill a tunnel from anteromedial or anterolateral tibia towards the desired fixation site at the posterior tibial plateau [21, 33]. Either retrograde or anterograde overdrilling of the guide was performed to establish the transtibial tunnel. The sutures were shuttled through the tunnel with a nitinol passing wire and then the meniscal root was reduced down towards the footprint through suture tensioning. Fixation was performed on the anteromedial or anterolateral tibia by tying the suture ends over a suture button (TightRope ABS®, Arthrex, Naples, US). The postoperative rehabilitation program consisted of non-weightbearing on crutches without brace-support for 6 weeks postoperatively, partial weight bearing for another 6 weeks and then gradually increased weightbearing until 4 months postoperatively. Knee bending of more than 90 degrees with load was prohibited until 3–4 months postoperatively [30].

### Outcomes

Demographic and surgical data was extracted from the patients journals: Age, gender, smoking habits, left or right knee, medial or lateral injury, concomitant injuries (ACL injury or other meniscal or cartilage injuries) and additional surgical procedures (ACL primary reconstruction or ACL revision procedures, additional meniscal or cartilage surgery) were recorded. Any reoperations of the affected knees were recorded.

### MRI

**Fig. 1:**
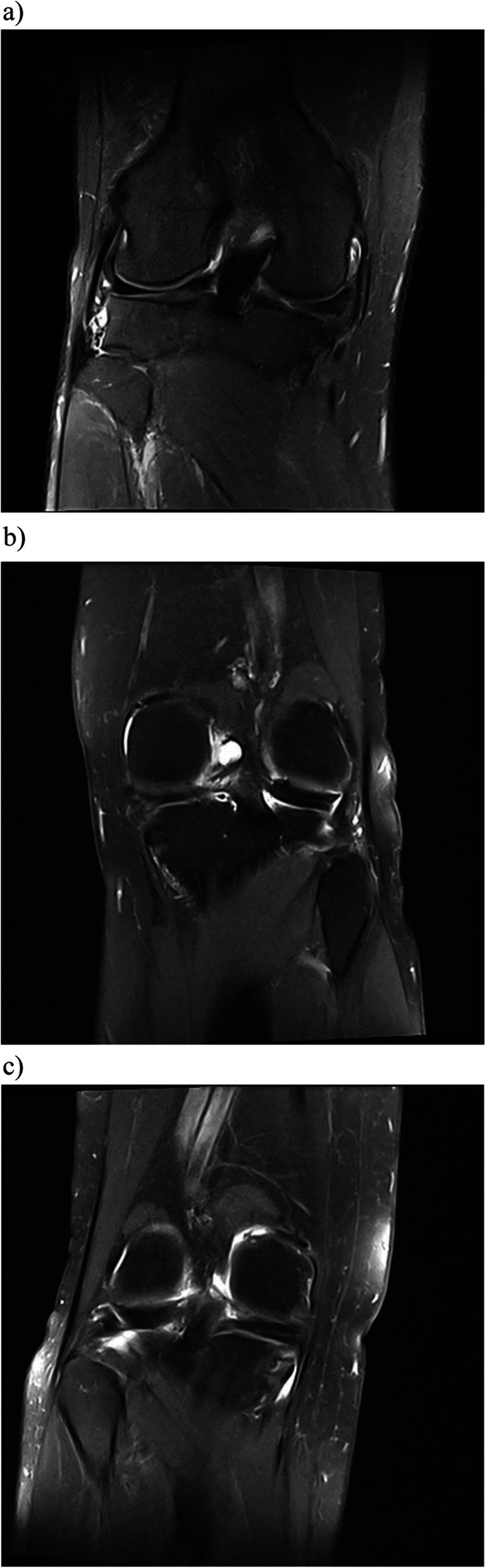
The figures present a (**a**) non-healed, (**b**) partially healed and (**c**) a healed medial meniscus posterior root tear (MMPRT) repair

The continuity/discontinuity of the menisci in all three planes (axial, coronal and sagittal) and amount of extrusion (mm) were used to evaluate healing after repair. An intact repair was only characterized by root continuity in all three planes, a partial repair was characterized by partial discontinuity in one or two planes (coronal, sagittal or axial plane), and an incomplete repair was characterized by complete discontinuity (Fig. 1) [18]. The meniscal extrusion pre- and postoperatively was measured at the broadest level of the eminentia medialis and lateralis in the coronal plane and reported in mm [25].

### Radiographic imaging

Anteroposterior radiographs of the knees were used to evaluate the degree of radiographic osteoarthritis according to the Kellgren-Lawrence (K-L) classification [16].

### Clinical outcomes

Each patient filled out patient reported outcome measures (PROMs) including the Knee Injury and Osteoarthritis Outcome Score (KOOS), Tegner activity scale and Lysholm score [34, 35]. The Global Rating of Change (GRoC) score was presented as a Likert scale from 0 to 5 (0 = very much better and 5 = very much worse). GRoC was measured for both function (GRoC Function) and pain (GRoC Pain) [15].

### Statistics

Demographic data were presented in counts and percentages for nominal variables and with means and ranges for the continuous variables. A paired t-test was used to compare the change of extrusion within each group, ordinal data was compared by the independent t-test or Mann-Whitney test. For comparison of between-group differences of categorical data a chi-square test or Fisher exact test was used. Significance level was set at 5 % (*p* = 0.05). To assess measurement reliability, the single measures, absolute agreement definition of the intraclass correlation coefficient (ICC) for inter-rater reliability was calculated with a two-way random effects model. All analyses were performed in IBM SPSS© statistical software Version 25. Post hoc power calculation revealed that a with 18 participants in each group and 1 mm difference in extrusion set as a clinical meaningful difference between the two groups, the study showed a power of 70% (*p* = 0.05, SD = 0.9 mm).

## Results

**Fig. 2:**
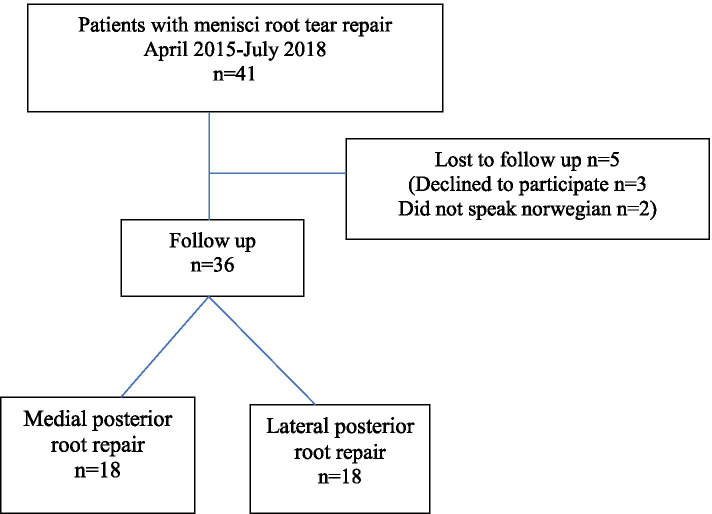
Flow chart posterior meniscal root repairs

Forty-one patients had a transtibial suture repair of their meniscal posterior root tear during the inclusion period. Thirty-six (88%) of the patients participated in the study (Fig. 1). The mean follow-up time was mean of 26 months (range 12 to 38 months). Eighteen patients had a medial posterior root repair and 18 patients a lateral posterior root repair. Baseline demographics and clinical characteristics of both patient groups are presented in Table 1. Most patients with LMPRTs were treated for a concomitant ACL rupture and in 11 of 18 an additional meniscal injury was found. Patients with LMPRTs had surgery at younger age and presented lower BMI than patients with MMPRTs. Cartilage degeneration (ICRS grade 1 or 2) was detected at surgery in 5 of the 18 LMPRT compared to 17 of 18 MMPRTs. (Fig. 2)

**Table 1:** Baseline demographics and surgical data of study patients with posterior meniscal root repairs for the medial and lateral meniscus

	Medial meniscus (*n* = 18)	Lateral meniscus (n = 18)	*P*-values
Age (median ± IQ range)	54.6 ± 15 (35–65)	26.3 ± 11 (18–45)	< 0.05*
Sex (male/female)	7/11	8/10	ns#
Side (right/left)	9/9	6/12	ns#
Body mass index (mean ± SD)	29.1 ± 4.7	25.3 ± 2.2	< 0.05*
ACL reconstruction(n)	0	17	< 0.05#
ACL revisions(n)	0	6	< 0.05#
Additional mensical injury(n)	0	11	< 0.05#
Follow-up (months)	26	25	ns*

^*^Students t-test/Mann Whitney test

^#^Chi-square/Fishers exact test

*ns* Non significant

### MRI

**Fig. 3:**
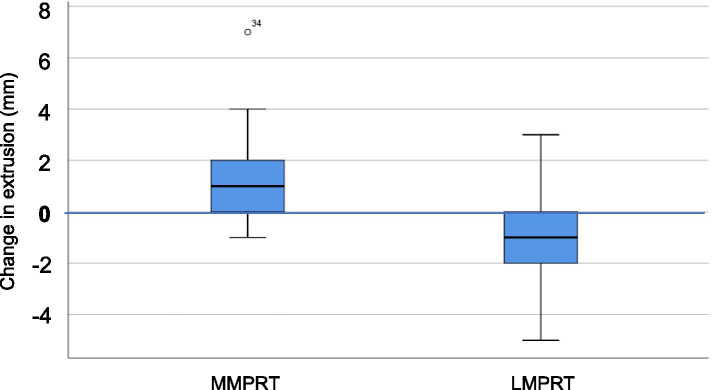
Change in meniscal extrusion (mm) from pre-to postoperatively for the LMPRTs (lateral meniscus posterior root tears) and MMPRT (medial meniscus posterior root tears)

The degree of healed, partially healed and incomplete healing of the LMPRTs and MMPRTs, classified by MRI are presented in Table 2. LMPRTs showed the highest amount of healing and the MMPRTs showed the highest amount of meniscal extrusion at follow-up. The change in meniscal extrusion measured from pre- to postoperatively are presented in Table 2. LMPRT repairs: MRI in 18/18 patients showed a mean preoperative meniscal extrusion at 2.3 ± 1.49 mm and a mean postoperative extrusion at 1.4 ± 1.09 mm (*p* = 0.08). In 11 of the 18 patients the rupture was classified as healed. MMPRT repairs: MRI from 18/18 patients showed a change in meniscal extrusion from 3.1 ± 1.55 mm preoperatively to 4.8 ± 1.90 mm postoperatively (*p* < 0.05). In 5 of these 18 patients the suture was classified as healed on MRI at follow-up. There was a significant difference between groups in meniscal extrusion from pre- to postoperatively (*p* < 0.05) (Fig. 3). The inter-rater reliability (ICC) of the MRI scores was measured to be 0.58 (CI 0.15 to 1.0, SEM 0.19) for measurements on continuity and 0.74 (CI from 0.59 to 0.89, SEM 0.06) for measurements on meniscal extrusion. (Table 3).

**Table 2:** MRI and radiographic findings of Meniscus posterior root repairs

	Medial meniscus. Root (*n* = 18)	Lateral meniscus root (n = 18)	*P*-value
**MRI:**			
Menisci healing status: complete/partial/non-healed (n)	5/8/5	11/5/2	ns#
Complete healing:	5	11	< 0.05#
Menisci extrusion change pre- to postop (mm ± SD)	1.50 ± 1.9	−0.94 ± 1.9	< 0.05*
**Radiographic imaging affected knee:**			
KL grade preop 0/1/2/3/4 (*n* = 2 missing)	6/9/1/0/0	10/5/3/0/0	ns#
KL grade FU 0/1/2/3/4	1/5/7/4/1	7/4/4/3/0	ns#

^*^Students t-test

^#^Chi square test/Fisher’s exact test

*ns* Non significant

**Table 3:** Patient reported outcome (mean ± standard deviation) for medial and lateral posterior meniscal root repairs at follow-up. (ADL, activity of daily living; QoL,quality of life)

	Medial root repair(n = 18)	Lateral root repair(*n* = 17)	*P*-value*
KOOS Symptoms (± SD)	76.6 ± 16.9	79.2 ± 15.1	ns
KOOS Pain (± SD)	70.5 ± 26.1	83.3 ± 15.8	ns
KOOS ADL (± SD)	79.3 ± 24.0	91.9 ± 11.1	ns
KOOS Sports (± SD)	47.2 ± 26.2	59.7 ± 17.1	ns
KOOS QoL (± SD)	53.5 ± 24.8	60.7 ± 23.7	ns
Lysholm (± SD)	74.4 ± 18.5	79.2 ± 16.0	ns
Tegner (± SD)	2.3 ± 2.1	4.3 ± 2.0	**< 0.05**
GRoC Function (± SD)	4.1 ± 1.2	3.5 ± 1.3	ns
GRoC Pain (± SD)	4.2 ± 1.3	3.3 ± 1.2	**< 0.05**

*Students t-test

### Radiographic imaging

Osteoarthritis according to the K-L classification of standard anteroposterior radiographs for the two groups are presented in Table 2. At follow-up the LMPRT group showed a mean change in K-L grade of 0.1 grade and the MMPRTs had a mean change of K-L grade from preoperatively to follow-up at 1.2 grades.

### Functional scores

No differences between the two groups were found for all KOOS subscales and the Lysholm score. The Tegner activity scale was higher and the GRoC Pain score lower in the LMPRT group compared to the MMPRT group.

### Reoperations

Four patients were reoperated due to complains related to the fixation button (1 MMPRT and 3 LMPRTs). Two MMPRTs were treated with high tibial osteotomy. One LMPRT patient had a partial meniscectomy and one was reoperated because of a cyclops lesion.

## Discussion

The main finding of this study was that the majority of LMPRTs repaired by a transtibial suture technique showed continuity and healing of the meniscal fibres at the attachment site and the meniscal extrusion was diminished, whereas the MMPRTs had increased extrusion and showed less tendency of continuity of the fibres at attachment site according to MRI (28% vs 61%). This study confirms that medial meniscus posterior root repairs are not able to restore anatomy back to normal state and the prevention of further degeneration may be postponed but not prevented by surgical intervention. Furthermore, this study confirmed that medial and lateral posterior meniscal root tears appear in different populations. Patients with LMPRTs were associated with traumatic injury and ACL reconstruction/revision reconstruction surgery. Patients with MMPRTs were associated with degeneration and progression of osteoarthritis and the patients were older and of higher BMI at operation.

The sustained extrusion of the medial menisci shown in this study, is in accordance with results from other studies: Chung et al. concluded that after repair, the meniscal extrusion was not reduced, and the technique did not prevent the development of OA [4]. In a systematic review of four different case-studies, Feucht et al. found that the healing was complete in 71 of 103 patients, partial in 29, and failed in 3. Meniscal extrusion however, decreased in only one of the four studies [8]. On the contrary, in LMPRTs, the outcome following repair has been found to be more promising: Okazaki et al. found a decrease in lateral meniscal extrusion following transtibial repair compared to other repair techniques and concluded with an increased chance to restore the hoop stresses in the menisci by this technique [29].

The diverging outcomes following MMPRTs compared to LMPRTs could have different explanations. First, most studies now reveal that there are demographic differences between the two groups. Krych et al. found that the LMPRTs were younger, had lower BMI, less cartilage degeneration and meniscal extrusion on MRI compared to patients with MMPRTs [20]. Secondly, patients with LMPRTs are often concomitantly treated with an ACL reconstruction. This could affect the outcome because the prospects of meniscal healing in general is improved when ACL reconstructions are performed in the same setting [19]. At last, lateral menisci with posterior root tears are less prone to excessive extrusion and the contact areas in the joint less diminished after LMPRT compared to MMPRT due to the meniscofemoral ligaments [2, 11]. Therefore a reduction of extrusion could be easier to achieve on the lateral side.  

Radiographic imaging showed a tendency towards progression of OA in the MMPRT group. To be able to conclude whether a repair might influence on OA ideally a control group, larger sample size and longer follow-up would have been of interest. Chung et al. found, in their metaanalysis on MMPRTs, that 35% of the patients with a MMPRT repair progressed according to the KL classification [4]. Pathological findings on MRI and radiographic imaging of the knee are not always correlated with clinical outcomes: Ulku et al. found a significant improvement in functional outcome after repair of MMPRTs even though a meniscal reduction was not achieved [36]. Other studies had similar findings for patients in the older age-group [5, 24].

The postoperative PROMs were similar in both groups, and in line with what has been reported in previously [8, 24]. The reason for a lack of distinction between the LMPRTs and MMPRTs in the functional scores could be because many of the LMPRT patients were exposed to additional surgery, even ACL revision surgery (6 out of 18 patients) at the time of root repair. Previous studies have reported significant change in IKDC and Lysholm score after LMPRT and MMPRT repairs, ACL revision-reconstructions though, are known to result in less favorable clinical and functional outcomes than primary reconstructions [26].

The ICC scores for continuity were considered moderate and the ICC for meniscal extrusion was considered good. A standardized MRI scanner and a specified protocol should ideally have been performed to improve the reliability for the measurements.

Limitations to this study were the retrospective study design and a low number of subjects with variable follow-up of the patients. The osteoarthritis development is highly time dependent and the results could have been affected by this, although both study groups presented similar average follow-up period. Limb alignment and residual knee laxity should also have been addressed as they are important factors affecting the healingpotential. Meniscal root tears treated with non-operative treatments or other repair techniques were not evaluated in this study and no control group was available. However, previous studies have shown less favorable results in patients with non-operative treatment or with partial meniscectomy [5, 6, 32]. Other limitations were the lack of baseline PROMs. A baseline score of the study participants could ideally have given a more specific picture of the treatment effect. The learning curve of the surgeons  is of concernwhen implementing a new technique, and this could possibly underestimate the treatment effect of the surgery. Finely, there is a lack of consensus on defining meniscal root healing on MRI, and a completely healed root repair was in this study only stated if continuity of the meniscus was confirmed in all three planes. Hence other studies might accept less strict criteria for healing compared to this study [20].

## Conclusion

Following transtibial suture technique, repairs of LMPRTs had a higher tendency of healing compared to MMPRTs. Furthermore, repairs of MMPRTs showed progression of the meniscal extrusion despite surgical intervention. The study also confirmed that medial and lateral posterior meniscal root repairs appear in different patient groups.
